# Telomerase Reverse Transcriptase (TERT) in Action: Cross-Talking with Epigenetics

**DOI:** 10.3390/ijms20133338

**Published:** 2019-07-07

**Authors:** Xiaotian Yuan, Dawei Xu

**Affiliations:** 1 School of Medicine, Shandong University, Jinan 250012, China; 2 Department of Medicine, Center for Molecular Medicine (CMM) and Bioclinicum, Karolinska Institute and Karolinska University Hospital Solna, 171 64 Solna, Sweden; 3 Shandong University-Karolinska Institute Collaborative Laboratory for Cancer and Stem Cell Research, Jinan 250033, China

**Keywords:** aging, cancer, epigenetics, telomerase, telomere lengthening, telomerase reverse transcriptase (TERT)

## Abstract

Telomerase, an RNA-dependent DNA polymerase with telomerase reverse transcriptase (TERT) as the catalytic component, is silent due to the tight repression of the *TERT* gene in most normal human somatic cells, whereas activated only in small subsets of cells, including stem cells, activated lymphocytes, and other highly proliferative cells. In contrast, telomerase activation via TERT induction is widespread in human malignant cells, which is a prerequisite for malignant transformation. It is well established that TERT/telomerase extends telomere length, thereby conferring sustained proliferation capacity to both normal and cancerous cells. The recent evidence has also accumulated that TERT/telomerase may participate in the physiological process and oncogenesis independently of its telomere-lengthening function. For instance, TERT is shown to interact with chromatin remodeling factors and to regulate DNA methylation, through which multiple cellular functions are attained. In the present review article, we summarize the non-canonical functions of TERT with a special emphasis on its cross-talk with epigenetics: How TERT contributes to epigenetic alterations in physiological processes and cancer, and how the aberrant epigenetics in turn facilitate TERT expression and function, eventually promoting cancer either initiation or progression or both. Finally, we briefly discuss clinical implications of the TERT-related methylation.

## 1. Introduction

Telomerase is a ribonucleoprotein elongating telomeric TTAGGG that repeats at the terminal of linear chromosomes. This ribonucleoprotein enzyme is composed of multi-subunits, but its core holoenzyme contains only a RNA template (TERC) and catalytic telomerase reverse transcriptase (TERT) [1,2,3]. TERC is ubiquitously expressed, whereas the *TERT* gene is stringently repressed in most human somatic cells, which consequently results in telomerase silencing [1,2]. Thus, TERT is the rate-limiting component to control telomerase activity. Physiological TERT induction and telomerase activation occur in small subsets of human cells, including embryonic and adult stem cells, activated lymphocytes, or other highly regenerative tissues, which play a pivotal role in stem cell biology, tissue homeostasis, and immune-modulation, eventually affecting the aging process [1,4,5]. The fundamental importance of TERT and telomerase in human beings is evidenced by the pathological alterations in patients carrying loss-of-function mutations of telomerase genes [6]. For example, the *TERT* gene mutation frequently causes hematopoietic stem cell exhaustion and bone marrow (BM) failure, as well as premature aging [6,7]. In late generation TERC- or TERT-knockout mice, stem cell functions are severely impaired, which in turn leads to degenerative defects, including BM aplasia and premature aging [4,8,9,10], while the TERT gene therapy rescued these mice [10]. Mechanistically, the wanting TERT and telomerase activity is unable to maintain telomere length required for sustained cell proliferation [8,9,10] because critically shortened telomere triggers telomere dysfunction and activation of the DNA damage response pathway, thereby inducing a permanent cell growth arrest so-called replicative senescence. Such telomere erosion similarly occurs progressively in dividing human somatic cells due to the lack of TERT/telomerase; and this is also the case in vivo with increased age [1,11]. The telomere erosion-mediated cellular senescence is believed to act as a key driving-force for aging and age-related degenerative diseases in human beings [1,3,11,12,13].

Replicative senescence resulting from shortened or dysfunctional telomere due to telomerase/TERT repression, although detrimental to tissue homeostasis and organ function, has been suggested as a trade-off for cancer resistance, a naturally occurring strategy against cancer evolved in long-lived and large-bodied mammalian species, including humans (with body mass >5–10 kg) [12,14]. For example, human somatic cells have telomere sizes between 8 to 20 kbs, only 1/10 to 1/5 long in cells from short-lived laboratory mice [3,12,14]. Moreover, these mouse somatic cells in general express TERT and telomerase activity. In accordance with the evolutionary cancer-resistance hypothesis, approximately one-quarter of aged people die of cancer, whereas this number can reach up to 90% in aged laboratory mice [14]. Because replicative senescence serves as a very potent barrier against unlimited cell proliferation, while infinite proliferation is a key hallmark of malignant cells [15], overcoming senescence barrier and acquiring an immortal phenotype by telomere stabilization is required for malignant transformation of human cells; and in most cases, this is achieved via TERT induction and telomerase activation [1,14,15,16]. Consistently, TERT expression and telomerase activity is detectable in up to 90% of human cancer-derived cell lines and primary tumors [1,2,16].

Given the widespread of TERT/telomerase activation and its critical role in malignant transformation, great efforts have been made to dissect how the *TERT* gene is de-repressed and telomerase is activated in cancer cells. The *TERT* is a single copy gene and harbors a single promoter with numerous binding motifs for various transcription factors (TFs) [17]. It was previously shown that the TERT promoter activity was intimately correlated with TERT mRNA expression—substantially higher in TERT/telomerase-positive cancer cells than in its negative normal counterparts, which indicates that TERT expression controlled at a transcriptional level represents an essential mechanism to activate telomerase in cancer cells [17]. The transcriptional regulation of the *TERT* gene occurs at multiple levels by various positive and negative factors or signaling pathways. The TERT promoter interacts with different TFs, responds to numerous physiological or oncogenic signals, and integrates these diverse and dynamic inputs to set the TERT mRNA output [2]. For example, the WNT/β-Catenin pathway directly activates *TERT* transcription and telomerase in both normal stem cells and cancer cells [18,19]. More recently, advances in high-throughput sequencing technologies have enabled comprehensive genetic characterization of the mechanism underlying TERT induction in various human malignancies [20]. The hotspot TERT promoter mutations lead to the generation of de novo ETS binding motifs, thereby stimulating TERT transcription and telomerase activation [2,3]. The structural variation including *TERT* gene rearrangements and onco-viral insertions have been identified as novel mechanisms to activate *TERT* transcription through enhancer hijacking [20,21,22,23,24,25]. Barthel et al [20] showed that *TERT* rearrangements frequently result in the direct overlapping between super-enhancers and juxtaposed *TERT* coding sequence, and such overlapping cause enhancer-hijacking through which massive chromatin remodeling and transcriptional activation of the *TERT* gene is achieved. In addition, the fine mapping of the TERT promoter methylation has identified TERT hypermethylated oncological region (THOR) as a cancer-associated epigenetic mechanism of TERT up-regulation in malignant cells (see below for details) [26]. Taken together, different mechanisms are employed by different types of normal and cancerous cells to activate *TERT* transcription and telomerase, and the elucidation of these mechanisms has significantly contributed to our in-depth understanding of TERT/telomerase regulation under both physiological and pathological scenarios.

**Figure 1:**
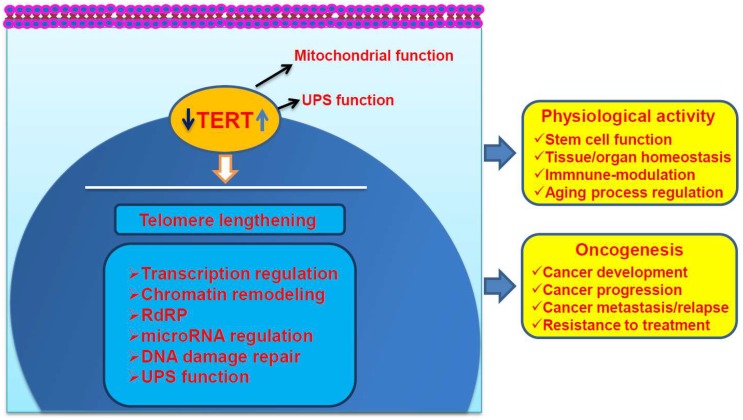
The telomere lengthening-dependent and independent functions of Telomerase Reverse Transcriptase (TERT). TERT/telomerase activation is required for both of physiological processes and transformation of human cells by stabilizing telomere length (telomere lengthening-dependent). The telomere lengthening-independent functions of TERT significantly contribute to both physiological processes and cancer initiation or progression, which include its effects on mitochondrial, ubiquitin-proteasomal systems (UPS), gene transcription, microRNA (miRNA) expression, DNA damage repair, and RNA-dependent RNA polymerase (RdRP) activity. TERT may shuttle between the cytoplasma and nucleus. The effect of TERT on UPS predominantly occurs in the nucleus but is also possible in the cytoplasma.

Telomere-lengthening is an established function of TERT/telomerase, but several lines of evidence have recently indicated that TERT exhibits multiple biological activities beyond its canonical action. TERT has been shown to modulate mitochondrial and ubiquitin proteasomal function, to regulate gene transcription and miRNA expression, to promote DNA damage repair, and to display the RNA-dependent RNA polymerase (RdRP) activity [8,27,28,29,30,31,32,33,34,35,36,37,38,39]. All these TERT effects are actively involved in physiological activities, eventually affecting aging process (Figure 1). On the other hand, these non-canonical activities of TERT drive cancer development and/or progression by conferring cancer cells survival, proliferation, stemness, invasive phenotypes (such as epithelial-to-mesenchymal, EMT), and many other features (Figure 1). Here we review the non-canonical functions of TERT with a special emphasis on its cross-talk with epigenetics: How TERT contributes to epigenetic alterations in physiological processes and oncogenesis, and how the aberrant epigenetics in turn regulate TERT expression and function, eventually promoting cancer initiation and/or progression. We will also discuss potential clinical implications of these findings.

## 2. The Function of Telomerase Reverse Transcriptase (TERT) in Cancer-Specific DNA Hypermethylation

### 2.1. The Aberrant DNA Methylation and DNA Methyltransferase (DNMT) Expression in Cancer

DNA methylation, perhaps the best-studied epigenetic modifications of DNA in human beings, is a covalent modification by adding a methyl group at position C5 of the cytosine ring in CpG dinucleotides [40]. There are up to 30 million CpG sites across the human genome and approximately 4,5000 CpG islands/haploid human genome [41,42]. CpG islands are typically present at or close to gene promoter regions/transcription start sites and in general hypomethylated in normal human cells [43]. However, aberrant DNA hypermethylation frequently occurs during oncogenesis, which leads to altered gene expression profiles and silencing of many tumor suppressor genes (TSGs) primarily through two well-characterized mechanisms [40,43,44,45]: First, the methylated CpGs within TF binding motifs in the promoter region may directly block the TF recruitment/interaction, inhibiting gene transcription; and second, the methylated promoter allures methyl CpG binding proteins that in turn recruit a variety of histone deacetylase complexes and chromatin remodeling factors, resulting in a condensed or inaccessible chromatin architecture and transcriptional repression.

DNA methylation is catalyzed by DNMTs that consist of at least three active members: DNMT1, DNMT3A, and DNMT3B [40,44,45]. They are canonical cytosine-5 DNMTs catalyzing the addition of methylation marks to DNA. In addition, DNMT3-like protein (DNMT3L), a non-canonical member, lacks essential components of the methyltransferase motif but binds to and regulates the activity of DNMT3A and DNMT3B [40]. DNMT1 shows a preference for hemi-methylated DNA and faithfully maintains the methylation state during DNA replication and cell division [40,44,45]. In contrast, DNMT3A and DNMT3B function as de novo methyltransferases due to their inability to differentiate between unmethylated and hemi-methylated CpG sites [40,44,45].

By modifying DNA methylation, DNMTs play an essential physiological role in various cellular processes, including embryonic development, cell differentiation, genome stability and DNA repair, and chromatin architecture or configuration [40]. The expression of DNMTs is highly regulated in normal cells. Consistent with their functional activities, DNMT1 expression is enriched in proliferating cells, while DNMT3A and DNMT3B are robustly expressed in embryonic and stem cells but down-regulated upon cellular differentiation. However, DNMTs are frequently dysregulated and aberrantly hyperactivated during malignant transformation [46], and their over-expression is responsible for the hypermethylation of panels of TSG promoters, thereby inactivating these TSGs in cancer cells, as described above. Such promoter hypermethylation-induced TSG silencing is critical to cancer development and progression, contributing to multiple cancer hallmarks [44,45,46]. The mechanism underlying cancer-related DNMT deregulation has been extensively explored but has remained incompletely understood.

### 2.2. TERT Activation of DNMT3B Transcription in Cancer

Interestingly, TERT and DNMT3 are expressed with similar temporal and spatial patterns during embryogenesis, development and cellular differentiation, whereas they are both up-regulated in malignant cells [1,2,44,45,46,47]. Furthermore, TERT over-expression in cancer cells leads to cellular resistance to DNMT inhibitor (DNMTi)-induced apoptosis, while its depletion sensitizes the DNMTi effect [38]. To evaluate their relationship in cancer, we examined the Cancer Genome Atlas (TCGA) dataset via cBioPortal [48,49] and observed a consistent positive correlation between TERT and DNMT3B expression in the majority of cancer types (Table 1). TERT expression was then manipulated in cancerous cells derived from hepatocellular carcinoma (HCC), and DNMT3B, but not DNMT3A nor DNMT1, was observed to be down- and up-regulated by TERT depletion and over-expression, respectively, which suggested the presence of a causal association between TERT and DNMT3B expression [37].

TERT has been shown to act as a transcription co-factor to regulate gene expression [33,50]. Typically, TERT interacts with TF Sp1, binding to the vascular endothelial growth factor (VEGF) promoter and facilitating *VEGF* transcription/cancer angiogenesis in human cancer cells and xenograph mouse cancer models [50]. *DNMT3B* is a target gene for Sp1 in human cells [51], and it is thus likely that TERT and Sp1 cooperate to activate the *DNMT3* transcription in a same manner. Indeed, TERT synergized with Sp1 to stimulate the DNMT3B promoter activity, and moreover, Sp1 inhibition significantly attenuated TERT-mediated DNMT3B up-regulation, while its over-expression restored DNMT3B expression in TERT-depleted cancer cells [37]. Importantly, the phosphatase and tensin homolog (PTEN) and Ras association domain family 1A, the two TSGs silent due to their promoter hypermethylation in these cancer cells, displayed corresponding alterations in their expression—up and down in TERT-depleted and over-expressed cells, respectively [37]. The pyrosequencing analysis revealed that a significant demethylation in the PTEN promoter occurred in TERT-depleted cancer cells, which was coupled with the DNMT3B down-regulation in these cells [37]. Thus, it is evident from these findings that TERT contributes to the aberrant PTEN promoter methylation by up-regulating *DNMT3* transcription in cancer cells.

It is well established that PTEN inhibits AKT (protein kinase B) phosphorylation or the activation of the PI3K/AKT signaling [52,53], and thus PTEN silencing mediated by the TERT-DNMT3B axis should be functionally important in carcinogenesis. The increased AKT phosphorylation and super-activation is observed in cancer cells over-expressing TERT, thereby promoting cellular proliferation, survival, and oncogenic potentials [54]. Indeed, inhibiting PTEN degradation by targeting ubiquitin E3 ligase WWP1 (a PTEN-interacting protein) has been shown to restore PTEN activity in cancer cells, leading to growth arrest and diminished oncogenic capability of these cells [55]. As cancer-specific DNA hypermethylation is known to silence many TSGs, the TERT-DNMT3B axis is expected to have much broader effects on cancer initiation and progression. In HCC patients, DNMT3B over-expression predicts shorter overall survival and metastasis-free survival by epigenetically regulating metastasis-associated protein 1 expression [44,56]. In addition, DNMT3B directly binds to the promoter of the *metastasis suppressor 1 (MTSS1)* gene and inhibits MTSS1 expression independently of its methylation-catalyzing function [44,57]. Taken together, TERT and DNMT3B form a network signaling involved in aberrant DNA methylation, AKT activation and many other oncogenic activities. TERT has long been suggested as an attractive target for cancer therapy [58], and it will be interesting to test if it will be possible to simultaneously inhibit all these three signaling cascades by targeting TERT, thus killing many birds with one stone.

## 3. The Function of TERT in Chromatin Remodeling and Gene Transcription

### 3.1. Nucleosomes, the switching defective/sucrose non-fermenting (SWI/SNF) Complex, and <ATP-Dependent Chromatin Remodeling

DNA is present in the form of nucleosomes by wrapping around a histone octamer consisting of histone proteins H2A, H2B, H3, and H4 in eukaryotic cells [59,60]. The nucleosome is a basic unit of chromatins and its compacted structure limits its accessibility, thereby stringently regulating gene transcription and expression [59,60]. ATP-dependent chromatin remodeling complexes are responsible for the disassociation of histone-DNA contacts and disruption of compacted nucleosomes. There exist at least four major types such complexes, including the switching defective/sucrose non-fermenting (SWI/SNF), the imitation-switch (ISWI), the Mi-2/nucleosome remodeling and histone deacetylation (NuRD), and the inositol 80 (INO80) [59,60]. These complexes in general contain multiple protein subunits contributing to the specificity in different biological contexts and one or two catalytic subunits utilizing the energy from ATP hydrolysis. For example, the SWI/SNF complex contains 12–15 subunits, depending on cell type and developmental stage, but always with a catalytic ATPase (either BRG1 or BRM), and three other core subunits (BAF150, BAF170, and SNF5) essential to the remodeling activity [59,60,61].

The SWI/SNF chromatin-remodeling complex plays an important role in multiple biological processes, including DNA replication, DNA repair and recombination, chromatin configuration, and gene transcription, among which is its transcriptional regulation as the most extensively studied function [59,60,61]. The SWI/SNF complex may act as either transcription activators or repressors in a context-dependent manner by interacting with both histones and DNA. It has been well established that the complex controls the transcription of sets of genes critical to early and late development, growth and cell differentiation/proliferation [59,60,61]. There is also strong evidence that the SWI/SNF complex activates the transcription of oncogenic factors whereas represses the expression of tumor suppressors, thereby promoting cancer development and progression [59,60,61]. One of the typical examples is the interaction between BRG1 and the WNT/β-Catenin signaling. The recruitment of BRG1 to the β-Catenin target gene promoters for chromatin remodeling is a prerequisite step for transcriptional activation, which is critical to the maintenance of cancer cell phenotypes and normal development of flies [62].

### 3.2. TERT Interaction withBrahma-Related Gene 1 (BRG)1 to Regulate the Transcription of β-Catenin Target Genes

In 2005, two research groups reported that TERT over-expression led to increased epidermal stem cell maintenance, activation and proliferation independently of its telomere-lengthening function [63,64]. To determine the underlying mechanism, Park et al searched for the TERT interacting proteins, and they found that the chromatin remodeling factor BRG1 was enriched in the TERT-containing complex [8]. Because BRG1 is required for β-Catenin-mediated transcription of target genes, whereas mouse skin-conditional over-expression of TERT highly mimics the effects of β-catenin over-expression on epidermal stem cells in mouse skin, it is likely that TERT exerts its function by interacting with BRG1 to facilitate the transcription of β-catenin targets. As expected, various molecular and cellular manipulations did demonstrate that TERT and BRG1 depend on each other to stimulate β-catenin-mediated promoter activation and target gene transcription, through which the β-catenin-regulated stem cell program of self-renewal, proliferation or survival are achieved [8]. Moreover, during *Xenopus* embryo development, TERT is similarly required for proper Wnt/β-catenin signaling and for formation of the anterior–posterior axis [8].

In support of the finding above, Chen et al observed that this identical mechanism was involved in the regulation of immunomodulatory properties of bone marrow mesenchymal stem cells (BMMSCs) [65]. TERT-deficient BMMSCs (TERT-/-BMMSCs) lose their capacity to inhibit T cells and ameliorate the disease phenotype in mice with systemic sclerosis, whereas TERT transfection in these cells rescues their immunomodulatory functions. Mechanistically, TERT, combined with β-catenin and BRG1, serves as a transcriptional complex, binding to the FAS ligand (FASL) promoter to up-regulate FASL expression, and thereby leading to an enhanced immunomodulatory activity [65]. Moreover, a significant improvement in the immunomodulatory capacity of normal BMMSCs could be achieved via the up-regulation of TERT expression [65].

In addition, Okamoto et al further identified the presence of the nucleolar GTP-binding protein nucleostemin (NS) in the TERT/BRG1 complex in CSCs [66]. NS is a self-renewal factor involved in stem cell self-renewal and proliferation [66]. The expression of each of these components is required to facilitate the CSC state, and these cells exhibit radio-resistance and robustly enhanced in vivo metastasizing. More recently, they showed that TERT regulated small RNA homeostasis, including both endogenous siRNA and miRNA, by interacting with BRG1 and NS. The depletion of either TERT or BRG1 led to dramatically diminished miRNA expression in cervical cancer and leukemia cells, to an extent even comparable to DICER inhibition-mediated miRNA down-regulation [27]. Because TERT over-expression or knocking-down affects miRNA precursors in addition to their mature miRNAs, TERT may regulate the miRNA transcription. The WNT/β-catenin, MYC and NF-kb are likely involved in TERT-modulated miRNA transcription, too [27]. Consistent with these findings in malignant cells, TERT was observed to regulate the expression of miR-1, miR-21, miR-29a and miR-208a in normal cardiomyocytes [67]. One of the functions for these small RNAs is to promote heterochromatin assembly and mitotic progression in a manner dependent on the RNA interference machinery [27]. However, it is currently unclear about the exact biological significance of TERT-mediated siRNA and miRNA expression. As miRNA dysregulation is widespread in cancer and significantly contributes to carcinogenesis, it is interesting to determine whether such TERT effect plays a part in cancer development and progression.

On the other hand, TERT was observed to directly interact with β-catenin and to stimulate the transcription of the β-catenin target genes. In gastric cancer-derived cells, TERT overexpression promotes, whereas its inhibition suppresses, CSC phenotypes and EMT, respectively [33]. Transforming growth factor (TGF)-β1 and β-catenin-mediated EMT was significantly attenuated by depletion of TERT expression [33]. Mechanistically, TERT is physically associated β-catenin, enhances its nuclear localization and transcriptional activity, and occupies the β-catenin target vimentin promoter. All these TERT effects were independent of its telomere-lengthening function or telomerase activity [33]. In prostate cancer cells, TERT is required for the symmetric CSC division by interacting with β-catenin and promoting β-catenin target expression. By doing so, TERT expands the CSC pool in prostate cancer [34]. Because EMT and CSCs are key factors promoting therapeutic resistance, aggressive diseases, metastasis and relapse in cancer, the identification of these TERT functions sheds light on its wide array of contributions to oncogenesis, which should have important biological and clinical implications.

However, Vidal-Cardenas et al [68] compared the gene expression patterns among wild type, TERC^–/–^ and TERT^–/–^ mice-derived cells, but they did not observe significant differences, which seems not to support the regulatory role of TERT in gene transcription. Because genetic compensation is a well-established phenomenon in which the loss of a gene can be compensated for by one or a set of genes, undetected phenotypic consequences of telomerase loss in these mice is likely due to a compensatory pathway masking the effects of the loss of TERT in the TERT^–/–^ mice [68]. To exclude this possibility, the authors further examined the gene expression in these mice-derived murine embryonic fibroblasts (MEFs) that experienced TERT loss at a very early stage, but again failed to detect differences in expression of Wnt/β-catenin targeted genes [68]. It remains unclear what cause such discrepant results. However, Vidal-Cardenas et al did not analyze the gene expression alteration in TERT-knockin mouse cells. TERT deletion and over-expression may affect stem cell function via different mechanisms. Flores showed that TERT knockout impaired stem cell function in a telomere lengthening-dependent manner, while TERT over-expression facilitated stem cell proliferation and self-renewal independently of telomere lengthening [63].

In addition, Listerman et al [69] investigated the relationship between TERT/BRG1 and β-catenin in human breast cancer cells, but failed to reveal a consistent TERT interaction with BRG1 and the Wnt/β-catenin pathway. It has been shown that BRG1 may serve as either a tumor suppressor or oncogene, dependent on cell and tissue types [70]. Furthermore, the SWI/SNF chromatin remodeling complex displays diverse activities in a context-dependent manner, and inactivation of different subunits frequently occur in human cancer [70]. These functional and genetic differences in cancer cell lines provide a plausible explanation for discrepant observations among different groups.

### 3.3. The Positive Feedback Loop Between TERT and TERT-Mediated Epigenetic Alterations

**Figure 2:**
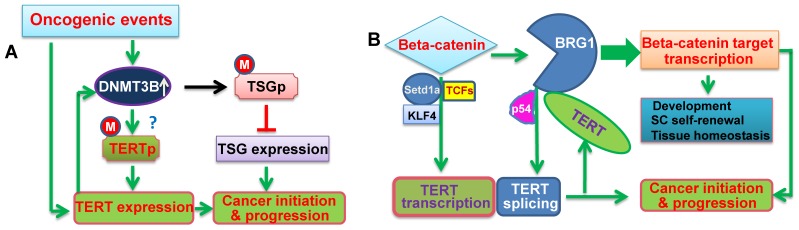
The schematic illustration of cross-talks between TERT and epigenetic factors. (**A**) The positive feedback loop between DNMT3B and TERT. Oncogenic events lead to the aberrant up-regulation of DNMT3B expression, thereby triggering the hypermethylation of the promoters in tumor suppressor genes (TSGs) and repression of these TSGs. On the other hand, DNMT3B over-expression may result in the TERT promoter hypermethylation, and consequently, activates *TERT* transcription. TERT induction in turn further up-regulates DNMT3B expression, which forms a positive feedback loop between them, and amplifies the oncogenic signaling to promote cancer initiation and progression. M: Methylated. ?: Not conclusive. (**B**) The positive feedback loop between β-Catenin and TERT. β-Catenin directly activates *TERT* transcription, while TERT acts as a co-factor to promote the transcription of the β-Catenin target genes by recruiting BRG1, which results in the formation of their positive feedback loop. In addition, BRG1 and P54 (nrb) cooperate to regulate TERT splicing and promote the generation of the full-length TERT mRNA. All these interactions play an important role in both physiological activities and oncogenesis.

As described above, TERT participates in important physiological processes and contributes to multiple cancer hallmarks by regulating DNA methylation and chromatin remodeling activity. On the other hand, these TERT-mediated epigenetic alterations in turn affect TERT expression and/or functions, thereby forming a positive feedback loop to amplify physiological and oncogenic signaling outputs (Figure 2) [71].

First, the TERT-DNMT3B axis may enhance *TERT* transcription and expression. It is well characterized that the *TERT* harbors a single promoter embedded in a CpG island (−1800 to +2300 relative to ATG), but the TERT promoter is unmethylated in normal human cells, whereas methylated in malignant cells [26,72,73,74]. Several lines of evidence suggest that the unmethylated promoter favors a repressor-binding [26]. Lee et al identified THOR as a cancer-associated epigenetic mechanism of TERT up-regulation in malignant cells [26]. THOR is a 433-bp genomic region encompassing 52 CpG sites located immediately upstream of the TERT core promoter. The hypermethylated THOR is a key TERT-upregulating mechanism in cancer, responsible for telomerase activation in 90% of human malignancies [26]. However, it remains unclear how the THOR hypermethylation occurs in oncogenesis. Because DNMT3B is responsible for de novo DNA methylation, its aberrant up-regulation during early oncogenesis may induce THOR hypermethylation through which the *TERT* gene is transcriptionally de-repressed. TERT induction in turn further facilitates the THOR hypermethylation, promoting cancer progression (Figure 2A). In addition, the TERT protein is a target for phosphorylation by AKT, and phosphorylated TERT exhibits much stronger activity [71,75,76,77], whereas the TERT-DNMT3B-induced *PTEN* gene silencing leads to robustly increased AKT function [37]. Conceivably, TERT and AKT amplify each other’s signaling outputs, thereby enhancing their oncogenic effects. In support with this view, TERT over-expression strongly increased the phosphorylation of AKT in leukemic cells, through which these cells became resistant to apoptosis induced by chemotherapeutic drugs and targeted therapies [54].

Second, β-Catenin directly activates the transcription of the *TERT* gene in both mouse embryonic stem cells and in human cancer cells [18,19]. β-catenin was shown to directly bind to the TCF site in the TERT promoter and to recruit the lysine methyltransferase Setd1a to the promoter region, while Setd1a, consequently, catalyzes histone H3K4 trimethylation at the promoter [18,19]. TCF1, TCF4, and KLF4 may be also involved in β-catenin-mediated *TERT* transcription. Given these observations, together with the effect of TERT on β-catenin target genes, a positive feedback loop between TERT and β-catenin may be readily present in stem and cancer cells. However, it is unclear whether BRG1 participates in the activation of TERT transcription by β-catenin, or whether TERT autonomously regulates its own transcription via interaction with β-catenin. Nevertheless, TERT and β-catenin may promote each other’s functional activities due to their common or overlapping effects on stem cell biology and carcinogenesis (Figure 2B).

Intriguingly, BRG1 was observed to be involved in the regulation of *TERT* transcription and mRNA splicing via a different mechanism [78]. Both BRG1 and BRM, the catalytic subunits of the SWI/SNF complex, are physically associated with p54(nrb), a human splicing family member protein, and PSF (polypyrimidine tract-binding protein-associated splicing factor). In BRG1-deleted cancer cells, BRM, p54(nrb), PSF, and phosphorylated RNA polymerase II are specifically co-localized in a region incorporating an alternative splicing acceptor site of TERT exon 7, which contributes to the generation of full-length TERT transcripts. However, BRM depletion led to down-regulation of TERT expression and an enhancement of ratios of exon-7-and-8-excluded TERT mRNA that encodes a β-site-deleted, inactive protein [78]. These cells underwent telomere shortening and eventual replicative senescence within two months. These data suggest that BRG1 or BRM, in concert with p54(nrb), is required to initiate efficient transcription and generation of full-length TERT transcripts by accelerating exon-inclusion, through which the levels of functional TERT protein and telomerase activity is enhanced. Thus, BRG1 actively up-regulates TERT expression and telomerase activity regardless of its interaction with β-catenin.

It is evident from all these findings that the interaction between TERT and epigenetic factors is very complicated. On one hand, they inter-depend on each other and integrate their signaling outputs to amplify their functional activities. On the other hand, they may exert their effects independently via different mechanisms. Nevertheless, all these activities promote the strong positive feedback loop formation, thereby resulting in synergistic effects on physiological processes and oncogenesis.

### 3.4. Clinical Implications of TERT Interaction with Epigenetics in Cancer

The cancer-specific TERT expression and telomerase activation has always aroused great enthusiasm in the potential clinical application of TERT/telomerase-based assays in the cancer field. However, a number of problems (such as unstable TERT mRNA and enzymatic activity) impede reliable utility of the direct TERT expression or telomerase activity assay as routine cancer diagnostic or disease monitoring tools. The hypermethylated TERT promoter has been shown unique to human cancer, as described above [55,72], and might serve as a diagnostic biomarker. We have recently identified two methylated CpGs in the TERT promoter region specific to tumors from patients with gastrointestinal cancer (GIC), and the methylated sites were detectable in stool from GIC patients, with sensitivity and specificity comparable to fecal occult blood test [72]. Lee et al revealed THOR spanning 52 methylated CpG islands in the TERT promoter, and assessments of all these 52 sites in stool are expected to further raise the sensitivity and specificity in GIC [55]. It was also shown that the methylated TERT promoter detection in cerebrospinal fluid could predict leptomeningeal metastasis [79]. These proof-of-concept studies suggest the feasibility of *TERT* promoter methylation analyses as a useful tool in noninvasive cancer diagnosis and progression surveillance.

In addition, the TERT promoter hypermethylation has been implicated in cancer prognostication. The association between the TERT promoter hypermethylation and poor outcomes or progression was reported in brain tumors, adrenocortical carcinoma, and other cancers [74,80]. The THOR determination is likely more powerful in survival prediction of cancer patients [55]. Taken together, the aberrant TERT expression and TERT promoter hypermethylation may serve as prognostic factors in multiple types of human cancer, and further evaluation including large cohorts of patients is required for future clinical application.

From the therapeutic point of view, targeting the interaction between TERT and epigenetics may contribute to the development of novel anti-cancer strategies. TERT inhibition results in the disruption of the TERT-DNMT3B and TERT-β-catenin loop signals, thereby exerting detrimental effects on cancer cells. On the other hand, telomerase inhibition can be achieved by targeting DNMT3B or β-catenin or both, or re-activating PTEN and other TSGs.

## 4. Conclusions

Human beings have acquired a robust cancer resistance strategy by repressing telomerase and maintaining shorter telomere over a long evolution period, however, such a cancer protective mechanism is a trade-off for aging/degenerations. Appropriate TERT/telomerase expression is required for stem cell biology, tissue homeostasis, and physiological aging, while the aberrant *TERT* gene de-repression/telomerase reactivation is essential to malignant transformation of human cells. Telomere lengthening is the canonical function of TERT or telomerase; however, the evidence has also accumulated that TERT displays much broader activities independently of its telomere elongation. The cross-talking between TERT and epigenetics leads to the interaction of TERT with key signaling pathways, including DNMT3B, PI3K-AKT, and WNT/β-catenin. These interactions are of importance for physiological and aging processes. In cancer, these cross-talks form positive feedback loops to amplify oncogenic signaling outputs. The disruption of these loops or networks by targeting TERT may be a novel anti-cancer strategy and are expected to be detrimental to cancer cells. Therefore, these mechanistic understandings of the TERT contribution to various cancer hallmarks should be helpful to the rational development of TERT-based tools for clinical diagnosis and management of cancer patients.

## Figures and Tables

**Table 1:** The correlation between telomerase reverse transcriptase (TERT) and DNA Methyltransferase 3B (DNMT3B) expression in the Cancer Genome Atlas (TCGA) cancer cohorts.

Cancer Type	N	Spearman Correlation	*p* Value
Hepatocellular carcinoma	360	0.25	1.254 × 10^−6^
Breast invasive carcinoma	994	0.33	6.38 × 10^−26^
Lung adenocarcinoma	507	0.46	1.38 × 10^−27^
Bladder cancer	412	0.20	3.300 × 10^−5^
Glioblastoma	273	0.49	1.34 × 10^−9^
Cutaneous melanoma	287	0.28	1.36 × 10^−6^
Colorectal adenocarcinoma	524	0.21	1.02 × 10^−6^
Prostate adenocarcinoma	491	0.22	1.087 × 10^−6^
Renal clear cell carcinoma	446	0.28	1.29 × 10^−9^
Pediatric neuroblastoma	1076	0.25	3.125 × 10^−3^
Endometrial carcinoma	507	0.30	4.17 × 10^−12^
Acute myeloid leukemia	165	0.317	3.29 × 10^−4^
Testicular germ cell tumors	144	0.748	1.43 × 10^−24^
Thymoma	119	0.566	1.90 × 10^−10^
Cervical squamous cell carcinoma	275	0.141	0.019

## Abbreviations

BMMSC	Bone marrow mesenchymal stem cell
CSC	Cancer stem cell
DNMT	DNA methyltransferase
GIC	Gastrointestinal cancer
HCC	Hepatocellular carcinoma
MTSS1	metastasis suppressor 1
NS	nucleostemin
RdRP	RNA-dependent RNA polymerase
SWI/SNF	Switching defective/sucrose non-fermenting
TERC	Telomerase RNA template component
TERT	Telomerase reverse transcriptase
TF	Transcription factor
TGF	Transforming growth factor
VEGF	Vascular endothelial growth factor
